# Supplementary material for: Mechanisms governing the pioneering and redistribution capabilities of the non-classical pioneer PU.1
Source: Nat Commun. 2020 Jan 21;11:402. doi: 10.1038/s41467-019-13960-2 (PMC6972792; doi:10.1038/s41467-019-13960-2)
Supplement: Supplementary file 7 — Source data [file 41467_2019_13960_MOESM7_ESM.zip › Source_Data/FigureS2/FigureS2B_MotifScanOutput/KmeansCluster5/homerResults/motif35.similar.html]

motif35

## Information for motif35

A
G
T
C
A
G
T
C
C
A
G
T
C
A
T
G
T
A
C
G
C
G
A
T
C
T
A
G
T
C
A
G
C
T
A
G
C
T
G
A
C
T
A
G
A
C
T
G
C
A
G
T
T
C
A
G
  
Reverse Opposite:  

A
G
T
C
G
T
C
A
G
T
A
C
A
G
T
C
A
G
C
T
A
G
T
C
A
G
T
C
A
G
T
C
C
G
T
A
A
G
T
C
G
T
A
C
G
T
C
A
C
T
A
G
T
C
A
G
  

|  |  |
| --- | --- |
| p-value: | 1e-34 |
| log p-value: | -7.981e+01 |
| Information Content per bp: | 1.755 |
| Number of Target Sequences with motif | 84.0 |
| Percentage of Target Sequences with motif | 2.11% |
| Number of Background Sequences with motif | 127.7 |
| Percentage of Background Sequences with motif | 0.30% |
| Average Position of motif in Targets | 96.5 +/- 75.5bp |
| Average Position of motif in Background | 108.9 +/- 63.7bp |
| Strand Bias (log2 ratio + to - strand density) | 0.3 |
| Multiplicity (# of sites on avg that occur together) | 1.01 |
| Motif File: | file (matrix) reverse opposite |

### Similar de novo motifs found

|  |  |  |  |  |  |  |  |
| --- | --- | --- | --- | --- | --- | --- | --- |
| Rank | Match Score | Redundant Motif | P-value | log P-value | % of Targets | % of Background | Motif file |
| 1 | 0.950 | A G T C A G C T C T A G T C A G C G A T C T A G C T A G T C A G C G T A C T A G A C T G A C G T C T A G | 1e-31 | -72.669418 | 2.36% | 0.44% | motif file (matrix) |
| 2 | 0.860 | T C G A A T G C G A T C G C A T C T A G T A C G C G A T C A T G C T A G C T A G C G T A T C A G | 1e-19 | -44.708846 | 2.91% | 1.01% | motif file (matrix) |
| 3 | 0.876 | A G T C A G T C A G C T C A T G C T A G A G C T C T A G C T A G C T A G G T C A C T A G | 1e-19 | -44.116885 | 1.93% | 0.50% | motif file (matrix) |
